# Cracking the genetic code with neural networks

**DOI:** 10.3389/frai.2023.1128153

**Published:** 2023-04-06

**Authors:** Marc Joiret, Marine Leclercq, Gaspard Lambrechts, Francesca Rapino, Pierre Close, Gilles Louppe, Liesbet Geris

**Affiliations:** ^1^Biomechanics Research Unit, GIGA in Silico Medicine, Liège University, Liège, Belgium; ^2^Cancer Signaling, GIGA Stem Cells, Liège University, Liège, Belgium; ^3^Department of Electrical Engineering and Computer Science, Artificial Intelligence and Deep Learning, Montefiore Institute, Liège University, Liège, Belgium; ^4^Skeletal Biology and Engineering Research Center, KU Leuven, Leuven, Belgium; ^5^Biomechanics Section, KU Leuven, Heverlee, Belgium

**Keywords:** Artificial Intelligence, genetic code deciphering, codon usage, codon embedding, deep neural network, data efficiency, natural language processing

## Abstract

The genetic code is textbook scientific knowledge that was soundly established without resorting to Artificial Intelligence (AI). The goal of our study was to check whether a neural network could re-discover, on its own, the mapping links between codons and amino acids and build the complete deciphering dictionary upon presentation of transcripts proteins data training pairs. We compared different Deep Learning neural network architectures and estimated quantitatively the size of the required human transcriptomic training set to achieve the best possible accuracy in the codon-to-amino-acid mapping. We also investigated the effect of a codon embedding layer assessing the semantic similarity between codons on the rate of increase of the training accuracy. We further investigated the benefit of quantifying and using the unbalanced representations of amino acids within real human proteins for a faster deciphering of rare amino acids codons. Deep neural networks require huge amount of data to train them. Deciphering the genetic code by a neural network is no exception. A test accuracy of 100% and the unequivocal deciphering of rare codons such as the tryptophan codon or the stop codons require a training dataset of the order of 4–22 millions cumulated pairs of codons with their associated amino acids presented to the neural network over around 7–40 training epochs, depending on the architecture and settings. We confirm that the wide generic capacities and modularity of deep neural networks allow them to be customized easily to learn the deciphering task of the genetic code efficiently.

## 1. Introduction

Artificial Intelligence (AI) can be used to try to unravel possible links in yet unexplored domains with the help of computers that are trained on data. The produced knowledge is data-driven and, in the best case scenario, the delivered knowledge is supported by a causal model and not by just a mere stochastic association. The results of this data-driven inference are sometimes difficult to interpret for human beings.

Deep Learning (DL) is a subset of Machine Learning (ML). Both DL and ML are part of the broad field of Artificial Intelligence. Deep Learning is more generic and more complex than Machine Learning. Deep Learning uses a generic layered structure of algorithms called an artificial neural network. The design of such a network is inspired by the biological neural network of the human brain, leading to a process of learning that is far more capable than that of the standard Machine Learning algorithms. The specific workflow in Machine Learning requires that relevant features most useful in describing the data for classification or regression purposes are first determined. Deep Learning skips the manual step of feature extraction and directly uses the raw data. However, this reduction in human intervention is offset by increased data needs and complexity. The *deep* in Deep Learning stands for the number of layers in the neural network architecture. The larger the number of layers, the deeper the neural network.

A large number of studies have combined omics (genomics, transcriptomics, and proteomics) and deep learning for years. Deep learning still holds great promise for genomic research due to its capacity of learning complex features in omics data and to infer complex relationship with responses (gene function, molecular structures, phenotypes, and disease outcome). A number of reviews on the application of Deep Learning in omics and in genome wide association studies (GWAS) have been published elsewhere (Li et al., 2018, 2019; Zhang et al., 2018; Eraslan et al., 2019; Munir et al., 2019; Rajkomar et al., 2019; Shen et al., 2022). DL technology has also been used with omics data in precision medicine applications together with information from medical imaging and clinical data (Martorell-Marugan et al., 2019). Whatever its successes in terms of prediction performance, applying DL technology to omics research or precision medicine still faces a number of difficulties. Two of them are (i) the “black box” problem and (ii) the data quality and availability problem. The black-box like algorithm obtained by deep learning cannot be understood or accepted by most people in the biomedical community. The self-learned algorithm which finally makes the predictions after training cannot help understand the mechanistic causal link between the data and the predictions. For example, in cancer gene expression profiles, the results of a cancer association to a gene expression profile do not explain why the gene profile expresses the cancer profile. In omics, this ability to explain is crucial for the biomedical research community (Zhang et al., 2018). The data quality and data availability problem is often underestimated by the community of end users having great expectations from DL technologies. The essence of DL is to learn rules according to the input data. The learning process requires huge amount of high quality curated data. Most of the data in omics are obtained through a large variety of experimental protocols. It is not guaranteed that the data are all produced under the same controlled conditions, are accurate, or are identically curated. Our study is a pedagogical contribution to address the black-box problem while controlling for the data quality and availability. One of our target audience is the biological research community. First, we think that our toy showcase provides a self-learned model after training, which will not be perceived as a black box. Indeed, the biological community can make immediate sense of the produced self-learned algorithm. The inferred rules were already known upfront, before they were re-deciphered by the Artificial Intelligence algorithm, as these rules happen here to be truly causal and simply reflect the textbook knowledge of the genetic code known to everybody. Our showcase should help the end-user community to gain trustfulness in DL technology. Our study aims at showing the audience that incorporating DL technology in biomedical research practice requires the research community to accept a trade-off between model complexity (or understandability) and the data efficiency (amount of data needed to produce the inferred rules with a chosen accuracy). The omics datasets that we used are very well-known to the biological research community audience. The toy showcase of the genetic code deciphering can be used has a benchmark problem to help the audience appreciate and assess the data size requirements when DL is applied to the field of omics. It also illustrates how using prior knowledge in the omics data structure compares with DL complexity (neural network depth and capacity) for improving the data efficiency to solve the problem at hand.

The genetic code is textbook scientific knowledge that was soundly established without resorting to Artificial Intelligence (AI). The purpose of this *Technology and Code* article is to showcase, using the genetic code as an illustrative toy project, how Deep Learning architectures can crack the code and unravel the correct knowledge. This showcase advocates the potential power and performance of self-learning algorithms. Additionally, our study aims at monitoring dynamically how the genetic code deciphering table is learned by the machine during the computer training.

Watson and Crick (1953) and others showed that nucleic acids (DNA and RNA) are the information-rich molecules that act as repositories and carriers of genetic information and that this information is encoded with only four nucleotides A, T, C and G in DNA (U replacing T in messenger RNA). Afterwards, it took 13 years for human researchers to understand how genes, with an encrypted message written with these four characters, could be translated into proteins for which there are 20 different amino acids characters. In those years, no sequence information was available. Using synthetic homopolymers like poly-A or poly-U, followed by copolymers with a defined sequence like poly-GUA as messengers, Nirenberg and Khorana progressively and laboriously cracked the genetic code, after Holley found the causal link (Nirenberg, 1968; Stryer, 1981). All three received the 1968 Nobel Prize in Physiology or Medicine. One of the salient feature of this translation mechanism is that it relies on chemical translators who speak the two languages i.e., nucleic acid (DNA/RNA) and protein languages. These translators, were discovered by Holley et al. (1965) and are called the transfer RNAs, tRNAs for short. The point is simply that two languages exist at the molecular biology level with molecules of two different chemical compositions. All living organisms use the two languages and use a lot of their energy to carry out translation, i.e., the synthesis of proteins. The genetic code is the universal translation rule mapping the sequence of characters for the sentence in one language (DNA/RNA) to the sequence of characters for the sentence in the other language (proteins). The sentences in both languages are made of words. In any natural language processing, the words are defined as N-grams (a particular sequence of N characters). The length N of the words are generally not specified. In the genetic code, the words in the source language (DNA/RNA nucleic acids) are trigrams (3-grams), i.e., three characters or one triplet of nucleotides. The words in the target language (protein language) are unigrams (1-grams), i.e., single amino acid characters. The mapping is not a bijection and the genetic code is said to be degenerate because multiple words (called synonymous codons) can map to the same amino acid word in the target language.

In this study, we built Deep Learning (DL) architectures to tackle this problem for which human beings already know the link. The goal was to check whether a deep neural network could re-discover, on its own, the link between codons and amino acids, and also maybe surprise us with its own representation of the data. Neural networks would not normally be used to decipher the genetic code. A more simple and direct approach of building and updating a dictionary through dynamic programming would provide a solution in an execution time shorter than tens of milliseconds and would not require a dataset with a large number of pairs of mRNAs with their associated proteins. Actually, the minimal dataset sufficient for the genetic code learning would be the list of the 64 mappings between codons and the amino acids. The structure of the dictionary linking codons to amino acids would have to be defined upfront. We, instead, want to resort to a self-learning process with as little human intervention as possible and we want to challenge and make use of the generic capacities of neural networks. Cracking the genetic code might appear to be a trivial task in natural language processing for a neural network. It is actually not so simple for three reasons. First, the number of classes in the target vocabulary is 21 (stop mark included), which is more than twice as much as the number of classes in the canonical MNIST digits classification problem, well-known to data and computing science engineers (LeCun et al., 1998; Deng, 2012). Moreover, in the MNIST digits classification, the inputs belong to a continuous 728-dimensional space while the input space in the genetic code deciphering problem has 64 discrete categories, making it more difficult. Second, both the input and target training datasets are inherently highly unbalanced due to the uneven distribution of codons within the transcript group and uneven distribution of amino acids within the protein group. Third, the purpose of cracking the code is not just to get a high accuracy translation, i.e., above 95%, according to state of the art common standards in ML/AI, but to determine the exact complete translation dictionary (stop codon included), which means an accuracy of 100%. The successful output of the training is to provide the full translation dictionary.

We compared the performance of different neural network architectures in cracking the genetic code upon training on the real human genome wide transcriptomic and proteomic dataset. For a given architecture, we varied the number of hidden layers (depth) and their capacity (width). Moreover, we investigated the effect of pre-processing the data on the networks performance. To that end, we compared the effect of the tokenization method (one-hot encoding vector size) and the effect of including or not a codon embedding layer for which we changed the dimension. For the different settings, we evaluated the cumulated minimal amount of data (number of codon/amino acid training pairs) required to decipher the code unequivocally, rare stop codons included, and to reach a training accuracy of 100% or an arbitrary small loss function. Additionally, we quantified how the use of the prior knowledge about the unbalanced amino acid frequency distribution could reduce this minimal data size.

## 2. Methods

The task of the neural networks that we implemented is to find the minimal dictionary that will map the set of the 64 unique triplets of contiguous characters (3-grams of nucleotides or codons) in the input space (DNA/RNA nucleic acid language) to the set of unique amino acid characters (unigrams of amino-acid characters) of the output space (protein language), including stop codons. The mapping will be a many-to-one to account for the degeneracy of the genetic code and the existence of synonymous codons. We compared different neural network architectures such as a fully connected multilayer perceptron (MLP), a Recurrent Neural Network (RNN) in its Elman network implementation (Elman, 1990; Amidi, 2019), its improved variants such as the Gated Recurrent Unit (GRU) and the Long Short Term Memory (LSTM; Hochreiter and Schmidhuber, 1997) and showed how they performed on discovering the genetic code. The MLP was chosen because it is the most fundamental neural network architecture. The RNN immediately comes to mind of data science engineers as soon as the input data are referred to as sequences.

*Training:* the models have been trained with transcript samples (mRNAs samples) as input data, and the predicted translated protein sequences (made up of amino acids) as output. The idea was to train the network on a mapping of mRNA sequences to their protein sequences and from there to map the codons to amino acids and get the neural network to find the correct decoding table.

*Input data:* we restricted ourselves to *Homo sapiens* data. The raw data used were downloaded from the public genomic repositories (Sayers et al., 2021), Ensembl (2022). The selected raw data are the whole human transcriptome i.e., all known mRNA sequences that are associated to approximately all 23, 000 human genes. These raw data have been pre-processed to extract only the full open reading frames (ORFs), meaning sequences that start with a “*start*” (AUG”) codon, end with a “*stop/non-sense*” (UAA”, UAG”, and UGA”) codon and entail a number of nucleotides which is an integer multiple of three. We did not include the so called 5′− or 3′− untranslated regions (UTRs) that are known to flank the ORFs respectively to the left and to the right. We also filtered out possible non-sense codon inserts or even possible inserts of a multiple of (three) independent single unread nucleotides, where frameshifts in the reading could be programmed and could occur. It must be emphasized here that we started from mature mRNAs (mature transcripts with exons only) for which alternative splicing had been fully achieved: we did not use immature mRNA sequences with introns, which are the intervening sequences in eukaryotic genes that are not translated.

*Output data:* for each and every ORF sample, we did associate synthetically the ground truth sequence sample of the translated mRNA, i.e., the produced protein.

We collected 69, 768 ORFs as input, and their 69, 768 protein sequence samples as output (ground truth). Both the input and output data are character sequences of variable lengths. The average size of a transcript is around 1, 200 nucleotides (characters), so the total number of nucleotides for which we have sequence information is around 83.7 million (or equivalently 27.9 million codons and thus around 27.9 million single amino acid characters). The redundancy in the available information was large enough and we had leverage to split the data into training and test sets. Due the nature of the problem addressed here, the test set and training set have very similar structures and we did not necessarily need a large fraction of the data to be used as test set. The input and ground truth output datasets are available on the GitHub project repository (github.com/MasterCube, 2022). We implemented a dataloader to split the data into a training set (90% sampling fraction) and a test set (10% sampling fraction). The input features and target features are fed to the models by batches (batch length = 64).

### 2.1. Implemented deep neural networks

**Figure 1:**
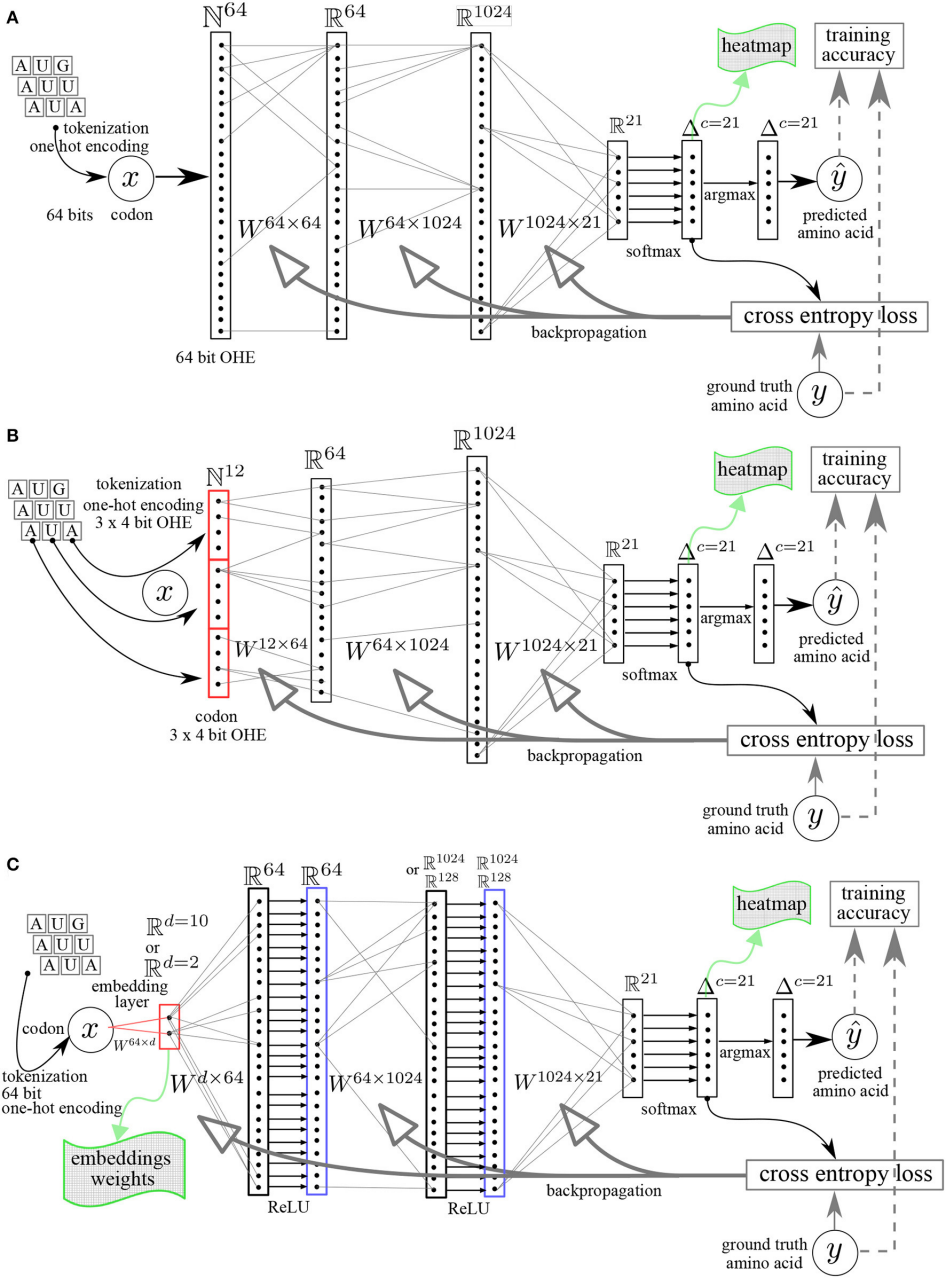
Multilayer perceptron (MLP) neural networks architectures. **(A)** Linear MLP with 64 bit OHE. **(B)** Linear MLP with 3 × 4 bit OHE. **(C)** MLP with embedding layer (dimension = *d*) and rectifying linear unit ReLU activation functions. The *Ws* are the matrices whose elements are the learnable parameters. *y*, ground truth value. ŷ, predicted value.

**Figure 2:**
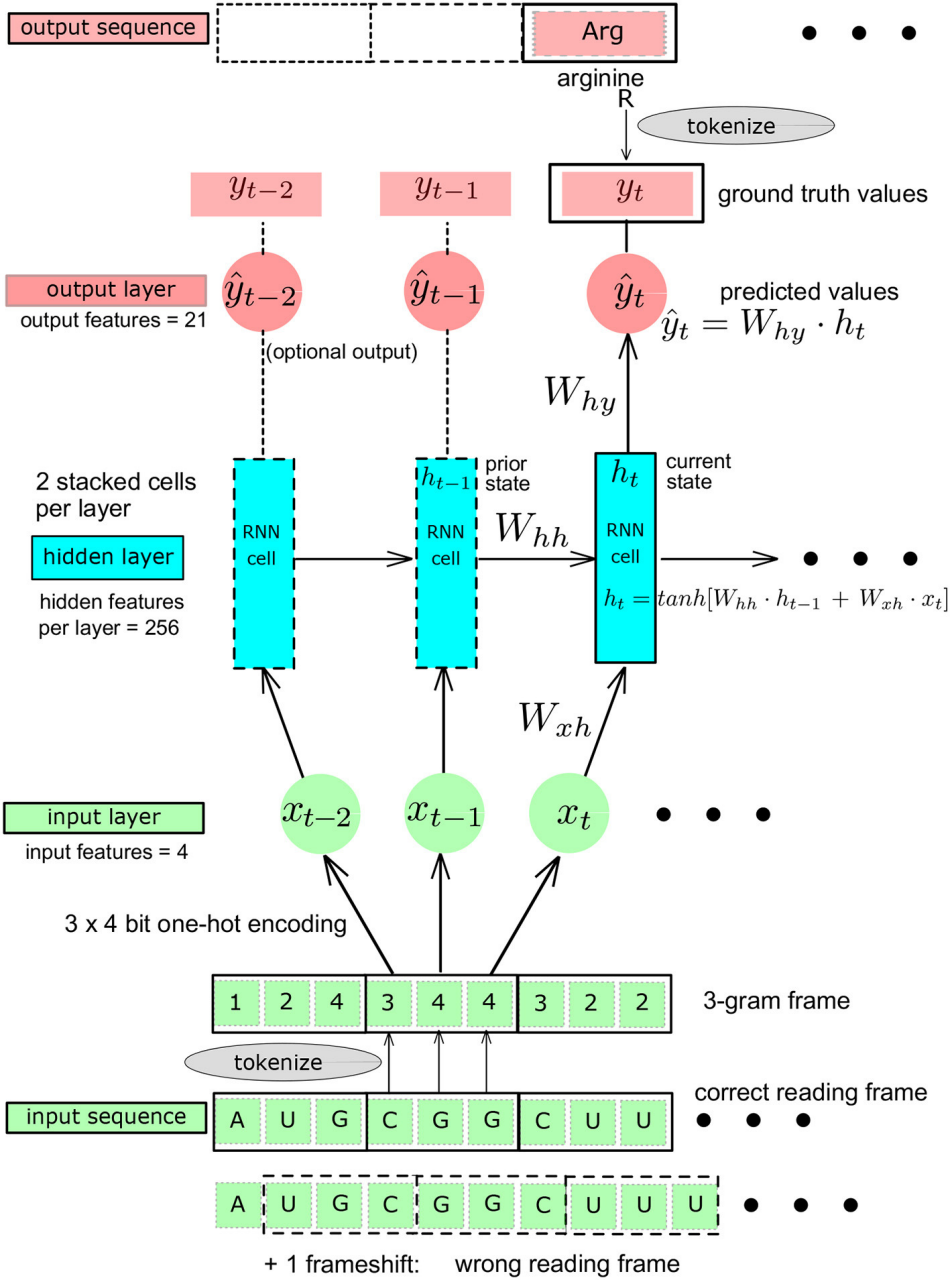
Schematic representation of the Recurrent Neural Network (RNN): baseline architecture showing a single RNN layer, adapted from Karpathy (2015). The *Ws* are the matrices whose elements are the learnable parameters. *y*_*t*_, ground truth value at *t*. ŷ_*t*_, predicted value at *t*.

The workflows are represented in Figures 1, 2 and entail a data pre-processing step (one-hot encoding), a codon embedding layer (optional) and the neural network layers with learnable parameters that will be optimized to minimize a loss function at each iteration on the training data.

To have our networks decipher the genetic code, we enforced the same assumptions as were historically stated by Nirenberg and coworkers (Nirenberg, 1968): (i) not less than three nucleotides are needed to code for the set of 20 amino acids (basic combinatorial calculus) and many *N* = 3−grams of nucleotides can map a single amino acid character; (ii) the code is non-overlapping and the 3-grams reading frame is provided and frameshifts do not occur; (iii) there are no punctuation marks inside the code; (iv) the first (three) nucleotides in each sequence form a “start codon;” (v) the last (three) nucleotides in each input sequence form a punctuation full stop mark i.e., a “stop codon.”

#### 2.1.1. Data pre-processing: One-hot encoding

*Tokenization and One-Hot Encoding (OHE)* convert the text characters to numerical values. This is required to allow the neural network to perform operations on numerical tensors representing the alphabet letter input data. Each unique character is assigned a unique numerical ID. When the tokenizer runs, it creates a word index (a numerical dictionary) which is used to convert each word as a vector of numerical values (a tensor). The length of the vector is the same as the length of the input sample. The One-Hot Encoding (OHE) is the process by which categorical variables (the four unique nucleotide characters A, U, C, and G) are converted into numerical values. The OHE of the alphabet of the nucleotide characters represents each nucleotide as a vector of four bits all set to zero except for a one corresponding to the alphabet index of the nucleotide character, e.g., the OHE of nucleotide A is (1, 0, 0, 0), U is (0, 1, 0, 0), C is (0, 0, 1, 0), and G is (0, 0, 0, 1).

We used two tokenization strategies for the one-hot encoding of the input data and we compared their impact on the training scores and the final accuracy.

*64 bit OHE*: OHE of each codon directly on 64 bits (Figure 1A). As there are 64 different codons, the codons vocabulary has a length of 64 and each word (codon) is indexed in this vocabulary set. The index is the position of the 1, all other elements in the vector are set to zero.

*12 bit OHE*: OHE of each codon as the concatenation of 3 nucleotides with a minimal OHE of the nucleotide alphabet, namely 3 × 4 bits = 12 bits (Figure 1B).

The amino acid ground truth data are always tokenized with a 21 bits OHE as the amino acid vocabulary has 20 amino acid plus one full stop mark.

#### 2.1.2. Codon embedding layer

The shortcoming of the one-hot encoding representation, besides just how huge it can get if the vocabulary size is huge, is that it treats all words (codons) as independent entities with no relation to each other. The OHE is chosen once and for all in the tokenization process. Codon embeddings, on the other hand, allow to incorporate semantic similarity in the words (codons): two synonymous codons coding for the same amino acid are more semantically similar to each other than to a codon coding for a different amino acid. The codon embedding process is similar to the one-hot encoding in that it maps the words to vectors but the vectors are dense (not sparse) and the vector elements can have any real values. The codon embedding can be part of the neural network and the embedding layer weights can be parameters of the model, i.e., the embedding weights are learnable parameters which can be trained. The embedding attributes are the vocabulary size and the dimensionality of the embeddings (word-embeddings tutorial, 2022). We investigated the effect of two embedding strategies on the performance of the network and investigated how similar the embedding weights' graphical representations are to the genetic code table. Two or more synonymous codons have a semantic similarity and should cluster together in the graph.

*Embedding dimensionality* *d* = 2. With two dimensions, the embedding features weights are tuples that can be represented in 2D and the Euclidean distance between the codons in the embeddings space directly reflects the semantic similarity and is easy to interpret.

*Embedding dimensionality* *d* = 10. With 10 dimensions, the embedding features may not have a direct interpretation but we still can represent the first pair of embedding weights graphically and investigate the learned semantic (2D projected) similarity.

The codon embedding layer included in the neural network is schematically represented in Figure 1C.

#### 2.1.3. Neural networks architectures

A feed-forward fully connected neural network, also called multilayer perceptron (MLP), could provide a first simple reference architecture to solve the problem, given that the codons are translated independently of each other once the 3-gram reading frame is known. A recurrent neural network (RNN) architecture allows to introduce a memory effect (or “sequential” effect) in the learning process (Karpathy, 2015; Amidi, 2019). We used this property in the genetic code learning architecture by implementing the successive reading of characters as unigram inputs, building a sequence length of 3 contiguous unigrams, defining a codon. The RNN produces an output only after a sequence of three nucleotide characters has been read (Figure 2). The RNN entails two stacked cells for which we compared two different sizes of the hidden layers: 64 and 256. To try to further improve the RNN architecture, we also used GRU and LSTM architectures. For each of these architectures, initialization and linearity were set as detailed below (Figures 1, 2).

*Initialization:* for the RNNs, GRU, and LSTM, we explicitly initialized the hidden cell states enforcing matrix orthogonality to prevent vanishing or exploding gradients issues.

*Linear or non-linear models:* for the fully connected MLP architectures, we used complete linear models, without any activation function except for the final Softmax needed after the last layer for the multinomial classification, where the Softmax is a non-linear multivariate function mapping any set of real numbers on a set of probability values all summing up to one (L1-norm). For the MLP with an embedding first layer, we used the rectifying linear unit, ReLU, compared to the non-linear hyperbolic tangent, tanh, as activation functions. The architectures of the neural networks are shown schematically in Figure 1. The number of hidden layers was varied from 1 (shallow) to 2 (deep) and the size (width) of the hidden layers was varied from 64, 128, to 1,024. This was done in order to increase the capacity of the neural networks and assess the impact of both depth and width on the data efficiency, i.e., the minimal amount of training data needed to reach an arbitrary small fixed value of the loss function or the complete deciphering of the genetic code. Figure 2 shows the RNN like architectures. The RNN was configured with two stacked cells (two layers) and with hidden size fixed to 64 or 256. By construction, the Elman architecture of RNNs, GRU, and LSTM entails hyperbolic tangent tanh activation functions and all these models are non-linear.

### 2.2. Loss function and learning metrics

The multinomial cross-entropy loss was the objective function to be minimized during training. The cross-entropy loss used the predicted probabilities for the 21 classes as the first simplex vector argument $p_{ŷ}\in \Delta^{c=21}$ and the ground truth simplex vector was used as the second argument $y_{\text{target}}\in \Delta^{c=21}$. The argmax was applied on the predicted probabilities for the 21 classes, $p_{ŷ}\in \Delta^{c=21}$, to obtain the predicted class as a new first simplex vector argument ŷ∈Δ^*c* = 21^ and the ground truth simplex vector, *y*_target_, was still used as the second argument. The accuracy (both training and test) was calculated by summing the dot product of these last two simplex vectors, i.e., ŷ·*y*_target_, within a batch, cumulating for the whole training set (or test set) and dividing by the training (or test) set sample size. The ultimate evaluation is to confirm the correct genetic code dictionary is discovered. The measure of success of the training is not as much in predicting correctly the protein target sequence as it is in providing the full translation dictionary that the learning process determined. The deciphered genetic tables were produced dynamically and monitored during training by tabulating a heatmap of the softmax output vector for all codons in a given batch, at each iteration, and for three different batches within each iteration. This was used to produce the dynamic heatmaps in the .gif format available on github.com/MasterCube (2022).

#### 2.2.1. Weights as optional argument

**Figure 3:**
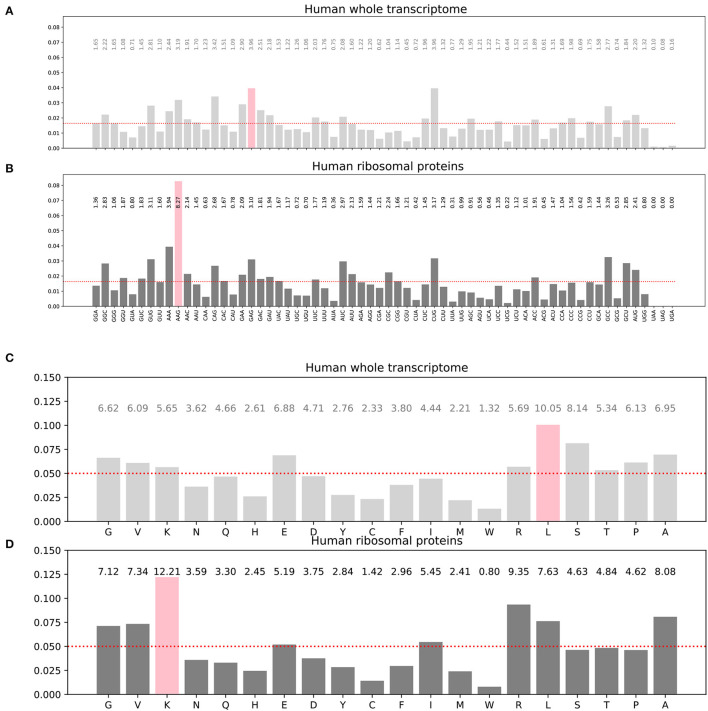
Histograms of the codon distribution in human transcriptome **(A)**. Codon frequency distribution in human ribosomal proteins transcripts **(B)**. Amino acid frequency distribution in all human proteins **(C)**. Amino acid frequency distribution in human ribosomal proteins **(D)**. Pink bars: most frequent codon in **(A, B)** or most frequent amino acid in **(C, D)**. The red dots show hypothetical uniform distributions for comparison with the observed distributions.

The frequency distribution of the codons and specifically the synonymous codons is not uniform in the input training dataset (Figures 3A, B). Similarly, the amino acid frequency distribution in the corresponding target dataset is not uniform either (Figures 3C, D). For instance, the histograms show that the most frequent codon in the whole transcriptome is GAG which codes for the glutamate amino acid (symbol E in single letter amino acid convention), even though glutamate is not the most frequent amino acid in the target proteome, while leucine is. Furthermore, these distributions are also protein family dependent. For instance, the histograms for the 76 human ribosomal proteins are different of the histograms for the whole human proteome as can been seen by comparing **(B)** to **(A)** and **(D)** to **(C)** in Figure 3. For the 76 human ribosomal proteins, the most frequent codon is AAG, and the most frequent amino acid is lysine K. As AAG codon codes for K, in this case, the most frequent codon and amino acid are matching. Transcriptome-wide or proteome-wide, this is generally not the case.

This inherent unbalanced property of the training set (also true for the test set) hampers the fair improvement of the genetic table dictionary upon training. Indeed, more frequent amino acids have a higher sampling rate, favoring the class of a highly abundant amino acids. For a rare amino acid, the learning is slower. To adjust for the unbalanced class distribution, it is possible to increase the loss function more when a minority class is misclassified compared to a majority class. To adjust for the underrepresentation/overrepresentation of the minority/majority classes, different weights (specific to each class) can be used to alter the loss function accordingly, so that the wrong predictions of rare amino acids are more penalized than for frequent amino acids.

We optionally incorporated weights in the inverse proportion of the class frequency distribution. The weight vector used was the inverse of the amino acid distribution known for the human proteome shown on Figure 3C.

We investigated how the weight option in the cross entropy loss function impacted the training profile, the final accuracy and the exhaustiveness of the genetic decoding table.

### 2.3. Optimization, learning rate, and number of iterations

The optimizer used in our training was the adaptative moment estimation (Adam) with a learning rate set to 0.05 (MLP) and 0.005 (RNN, GRU, and LSTM). The number of epochs (iterations) was set in the range 10–50. We did not use GPU/cloud computing allocation.

## 3. Results

**Figure 4:**
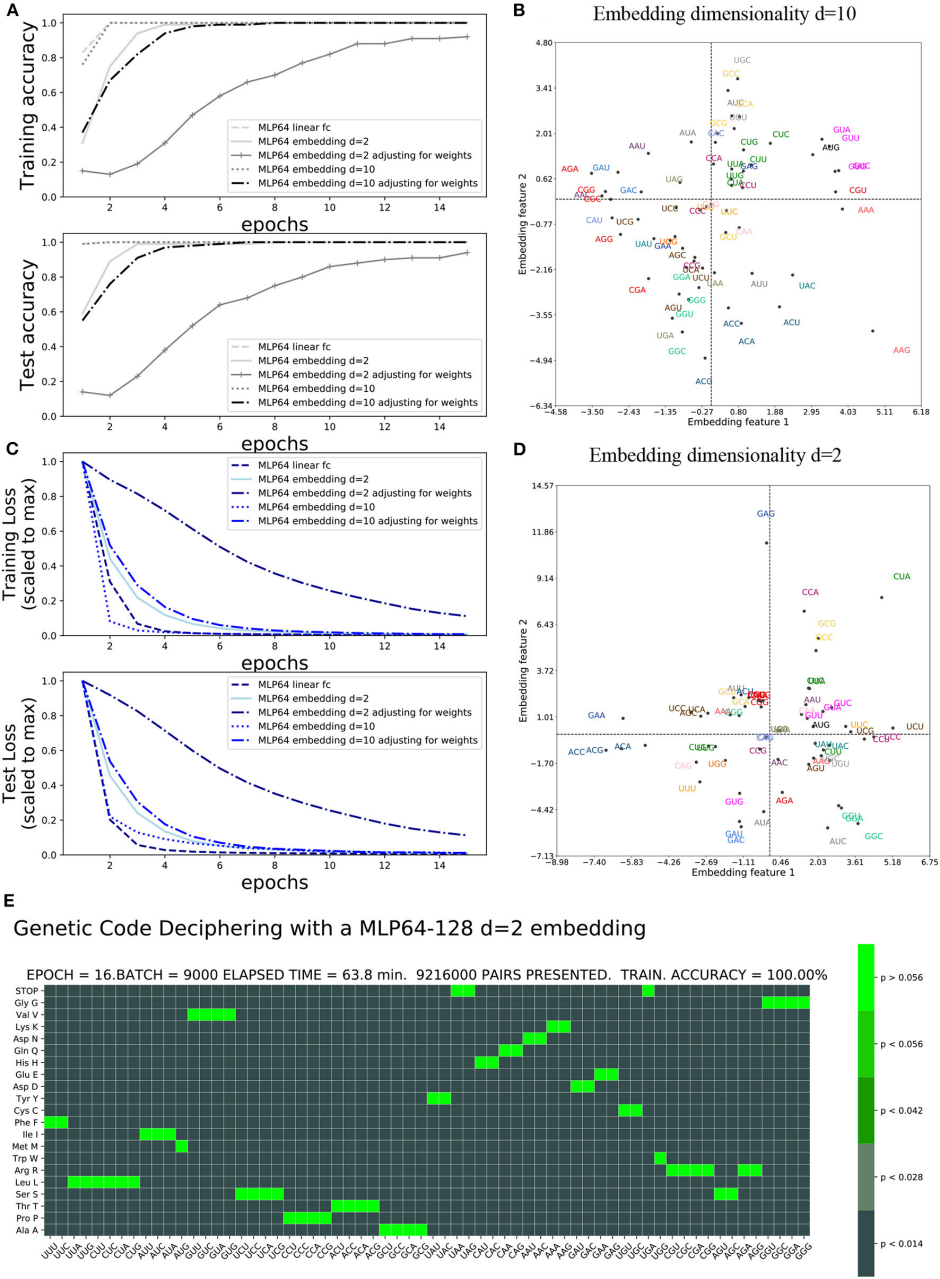
Impact of embedding layer and dimensionality on accuracy and loss: **(A)** Training and test accuracies. **(B)** Training and test losses. **(C)** *d* = 10 embedding first two features after 40 epochs. **(D)** *d* = 2 embedding features after 40 epochs. **(E)** Genetic code table as deciphered at epoch 16 for MLP OHE 64 bits with two hidden layers of size 64 and 128 and with codon embedding layer *d* = 2.

**Figure 5:**
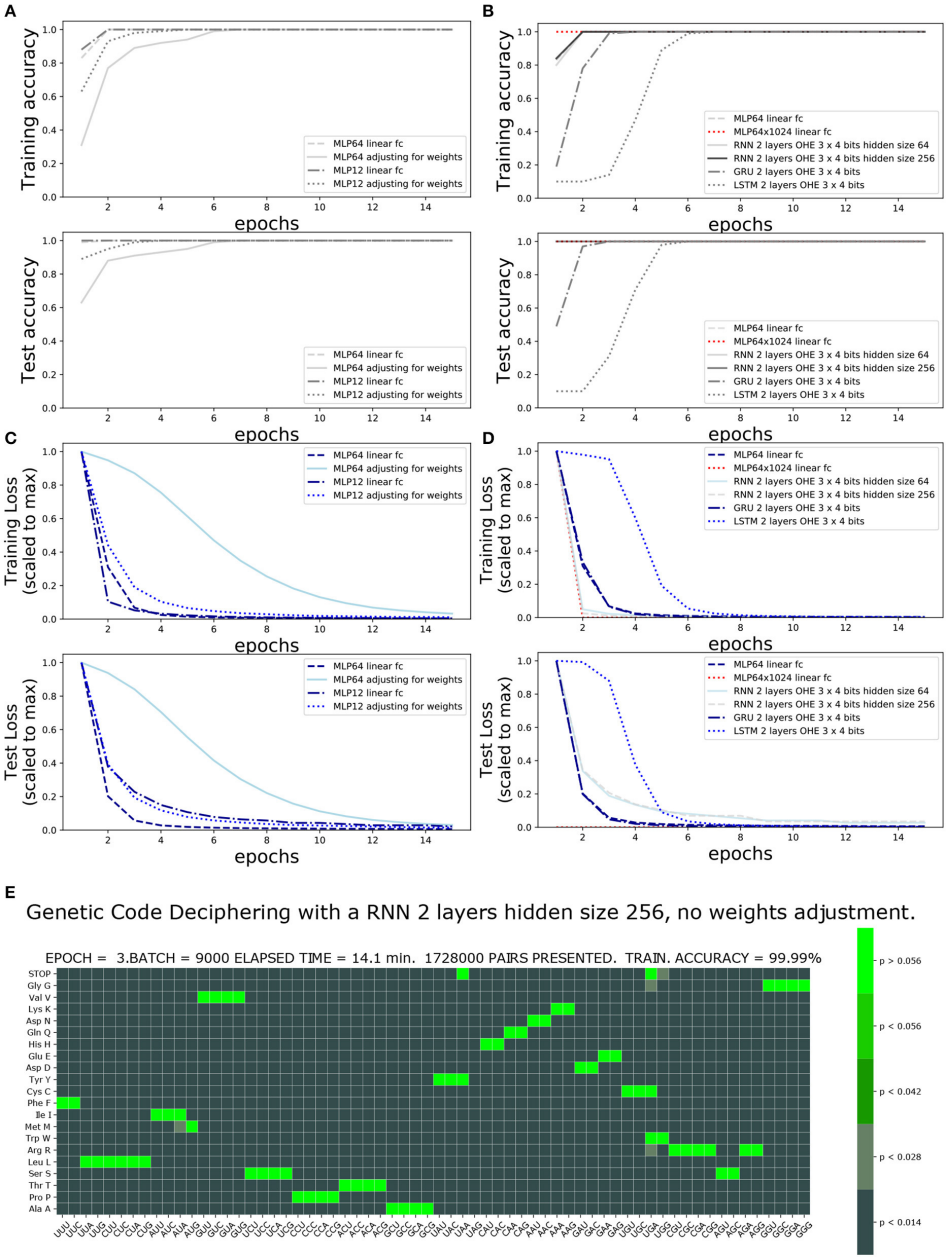
Impact of one-hot encoding size and network architecture on training accuracy and loss. One-Hot encoding 64 or 3 × four bits with and without weights adjustment: **(A)** Training and test accuracies. **(B)** Training and test losses. **(C)** Training and test accuracies comparing architecture and hidden size. **(D)** Training and test losses comparing architecture and hidden size. **(E)** Genetic code table as deciphered at epoch 3, for RNN two stacked layers of hidden size 256. Note that the stop codons (UAA, UAG, and UGA), tryptophane (UGG), cysteine, and tyrosine have not yet been unequivocally deciphered.

The accuracy and loss functions are shown in Figures 4, 5 for different settings and different neural network model training and testing. The elapsed computing times on a single GPU NVIDIA Quadro P320 were in the range of 9 min to more than 3 h per training, depending on the neural network architecture and the number of epochs. The fastest training was obtained with the complete linear multilayer perceptron with or without a codon embedding layer. When codon embedding was implemented, the best performance was achieved with the linear rectifying activation function ReLU. The dynamic heatmaps showing the updates of the genetic code deciphering table during training are available on github.com/MasterCube (2022). Direct links to seven instances of the dynamic heatmaps are provided here (Supplementary Videos 1–7). Figure 4E shows an instance of an heatmap at epoch 16 that was obtained for the two hidden layers MLP for which the codons were tokenized with 64 bits one-hot encoding and with an embedding layer of dimension *d* = 2. Figure 5E shows the heatmap at epoch 3 that was obtained for the RNN with two stacked hidden layers of size 256. This instance of the heatmaps shows that, at epoch 3 of the training process, the stop codons have not been unequivocally deciphered yet, although the decoding accuracy is already 99.99%. UAA is confused between a stop codon and tyrosine, Y. UAG has not been deciphered and UGA is confused between a stop codon and two rare amino acids cysteine, C, and tryptophane, W.

Table 1 compares, for the different architectures and settings, the number of training iterations (epochs) and the minimal data size required at training to achieve a complete and unequivocal genetic code table deciphering, i.e., including the rare stop codons UAG, UAA, and UGA. The architecture unequivocally deciphering the genetic code with the smallest amount of training data was the deep MLP having two hidden layers (64 and 1,024 in size) without activation functions, see first line in Table 1. The same architecture with the extra codon embedding layer of dimensionality *d* = 10 was the second best performing architecture without adjusting the unbalanced amino acid frequency distribution. However, the latter neural network architecture outperformed all other architectures after amino acid weights adjustment, see right column in Table 1.

**Table 1:** Minimal training data size in codon/amino acid pairs required for a neural network architecture to achieve complete unequivocal genetic table deciphering.

**Neural network architecture**	**Without weights adjustment**			**With weights adjustment**		
	**Ep.**	**GPU time** **(min.)**	**Data pairs** **Codon/aa**	**Ep.**	**GPU time** **(min.)**	**Data pairs** **Codon/aa**
**MLP with one-hot encoding, depth and width**						
MLP64 2 hidden layers 64 × 64 × 1024 × 21	8	14.8	4, 032, 000	38	71.4	21, 312, 000
MLP64 1 hidden layer 64 × 64 × 21	40	64.5	22, 464, 000^*^	24	40	13, 248, 000
MLP12 2 hidden layers 64 × 64 × 1024 × 21	18	35.7	10, 368, 000	7	13.5	3, 456, 000
MLP12 1 hidden layer 64 × 64 × 21	39	71.7	21, 888, 000	24	44, 8	13, 248, 000
**MLP with codon embedding layer and width**						
MLP64 *d* = 2,2 × 64° × 64° × 21 (tanh)	40	158.4	22, 464, 000^*^	40	159.5	22, 464, 000^*^
MLP64 *d* = 2,2 × 64^•^×128^•^×21 (ReLU)	17	63.8	9, 216, 000	9	27.0	4, 416, 000
MLP64 *d* = 10,10 × 64° × 64° × 21 (tanh)	30	179.0	16, 704, 000	34	196.0	19, 008, 000
MLP64 *d* = 10,10 × 64^•^×1024^•^×21 (ReLU)	17	129.3	9, 216, 000	2	9.9	384, 000
**RNNs with hidden cells states and size**						
RNN^†^ 2 hidden layers 12 × 256 × 256 × 21	22	77.6	11, 904, 000	17	60.9	8, 832, 000
RNN^†^ 2 hidden layers 12 × 64 × 64 × 21	22	77.8	12, 096, 000	30	100.4	16, 704, 000
GRU^†^ 2 hidden layers 12 × 64 × 64 × 21	29	103.7	16, 128, 000			
LSTM^†^ 2 hidden layers 12 × 64 × 64 × 21	40	150.4	22, 464, 000^*^			

A * superscript indicates that the rare stop codons are still not unequivocally deciphered at this training epoch. ◦ A tanh activation function is included in each hidden layer. • A ReLU activation function is included in each hidden layer, see Figure 1C. † RNN, GRU, and LSTM have two stacked cells. Weight adjustment was not investigated for GRU or LSTM.

### 3.1. Impact of codon embedding and graphical interpretation of codon embedding features

The incorporation of the codon embedding layer with dimensionality *d* = 10 allowed the MLP to achieve an unequivocal deciphering after 30 epochs, to be compared to the more than 40 epochs required for the completely linear MLP without embedding layer, with a single hidden layer of limited capacity of size 64. The dimensionality *d* = 2 was not sufficient to unequivocally decipher the rare stop codons after 40 epochs even with weights adjustment. Extending the capacity of the hidden layer to a size of 128 or 1,024 and with rectifying linear activation functions replacing the non-linear hyperbolic tangents, the stop codons were deciphered after 17 epochs. Adjusting for weights further improved the genetic code deciphering performance as the code was cracked after nine epochs (size = 128) or even two epochs (size = 1024).

These results show that a deeper network had a much better data efficiency to crack the code than a shallow network: the cumulated number of codon/amino acid data training pairs was reduced by more than 80%, from 22.5 to 4 million data pairs, when the fully linear MLP had two hidden layers instead of one and with a hidden size of 1,024 instead of 64. Additionally, the non-linear activation functions, tanh, were squashing the gradients and slowed down the deciphering of the code. As expected, the rectifying linear unit activation function prevented vanishing gradients to occur. Finally, adjusting for the amino acid frequencies' unbalanced distribution was more profitable for deeper fully linear networks (more hidden layers) or having larger hidden size, than for non-linear shallow networks.

The embedding features plot is shown for the *d* = 2 dimensionality embeddings and hidden layer capacity of size = 128 (Figure 4D). It is compared to the genetic code table that was deciphered at epoch 16. The semantic similarity of the synonymous codons that are correctly deciphered are indeed close to each other in the embedding features plot. For instance, GAU and GAC (blue) are the two synonymous codons for aspartate, D, and are close to each other in the plot. GGG, GGA, GGC, and GCU (light green) are the four synonymous codons for glycine, G, and are clustered colinearily in the plot. The first two embeddings features are shown after training at epoch 40 for the embedding dimension *d* = 10 and hidden layer capacity of size 1,024 (Figure 4C). With a complete unequivocally deciphered genetic code table after 17 epochs, the obtained embedding features' graphical representation, at the end of the training, can be considered trustful. For instance, arginine R has been exhaustively and exclusively decoded by the six codons CGU, CGC, CGA, CGG, AGA, and AGG (red). Except for CGU, these codons are colinearly clustered at the left most edge of quadrant 2 and 3, even in the 2D projected space out of the *d* = 10 dimensions. CGU, however, is located at the right edge of the plot. The interpretation of the embedding features plot with *d* = 10 dimensionality is more difficult for all codons as compared to *d* = 2 dimensionality.

### 3.2. Impact of OHE and class weight adjustment of the loss function on training scores

The impact of the one-hot encoding OHE settings is shown in Figures 5A, B. Comparing the fully connected linear MLP with 64 bit OHE with the fully connected linear MLP with 12 bit OHE shows that the training loss decreases faster for the OHE in 12 bits than in 64 bits. For the test loss, however, it is the opposite. A training accuracy of ~100% is achieved within two epochs in both cases. This ~100% excellent accuracy is only apparent because the training set did not necessarily include rare stop codons within these two epochs. An unequivocal genetic code table deciphering, including the rare stop codons, is achieved only at epoch 39 for the 12 bit OHE and over epoch 40 for the 64 bit OHE (Table 1). The neural network keeps learning even after a 100% accuracy was reached because the optimizer keeps minimizing the cross entropy loss. Figures 5A, B furthermore show that the weight adjustment compensating for the uneven distribution of the amino acid classes dampens the decrease in the training and test losses and the accuracy increase rate. It is worth noticing that without weight adjustment, the misclassifications in the deciphering of the genetic code occur more often on the stop mark * (codons UAA, UAG, and UGA) or on the rare amino acids such as methionine (M, AUG) and tryptophane [W, UGG; Figure 5E and dynamic heatmaps in GitHub project repository (github.com/MasterCube, 2022)]. For networks with limited layer capacity, the stop codons are better (sooner) resolved upon weight adjustment as can be observed on the heatmaps for the last epochs (Table 1). The target classes weight adjustment reduced the minimal required training data pairs by 40 to 65%. In the context of this study, for a very high capacity neural network, the weight adjustment is not beneficial as can been seen by comparing the two main columns in the first line of Table 1.

Using the prior knowledge of the unbalanced representation of amino acids in the training dataset and in the human transcriptome and proteome in general significantly shrinks the required amount of training data to achieve a complete deciphering of the genetic code by a fully linear multilayer perceptron (with 64 or 12 bit OHE) as can be seen by comparing the two main columns in Table 1. Indeed, both with the 12 or 64 bit OHE, the total minimal number of codon/amino acid pairs to be presented to the MLP neural network to completely crack the code decreases from ~22.4 million to ~13.2 million. Similarly, with the codon embedding layer and the linear activation function, ReLU, where it decreased from ~9.2 million to ~4.4 million and even 0.38 million.

### 3.3. Impact of architectures

The performance of the four architectures (MLP, RNN, GRU, and LSTM) is compared in Figures 5C, D and in Table 1. The training loss decreases faster with the deep MLP with two hidden layers of size 64 and 1,024 than with the RNN having two stacked cells layers of size 64 or 256, or with the MLP with a single hidden layer of size 64. A 100% accuracy both for training and testing is reached within two epochs. The GRU and LSTM require four and six epochs to reach similar accuracies.

The best performance in cracking the genetic code with the minimal amount of training data is achieved by the deep MLP with two hidden layers of size 64 and 1,024. Indeed, the complete genetic code is already deciphered after eight epochs, when only 4 millions of codon/amino acid data pairs have been presented to the network, and without using weight adjustment. The hidden state of an RNN cell allows to incorporate a memory effect while processing the codon input sequence. For instance, two of the rare stop codons UAA and UAG start with UA and they share this feature with only two other codons, UAU and UAC, coding for the single amino acid, tyrosine (Y). Having noticed this and the fact that an RNN can keep the memory of the first two nucleotides, it can discriminate earlier between a reduced subset of target amino acids during the learning process. This sequenced memory ability of the RNN is valuable for the deciphering of all codons. It comes at a price of a longer GPU execution time (time complexity) but allows to achieve the deciphering of the code upon training with almost only half as much the amount of training data, when compared to a classical fully connected multilayer perceptron having similar depth and size.

The forget gate specific feature of GRU and LSTM and the additional cell state effect that can be brought about by the GRU and LSTM appear not to be valuable for the decoding task of the short sequence within a triplet (codon). In general, for this particular genetic code deciphering task, the RNN, GRU and LSTM suffer from the presence of the hyperbolic tangent as imposed activation function. The non-linear activation function shrinks the gradient of the loss with respect to the learnable parameters and the rate of learning is slower. This hyperbolic tangent does not appear to be a smart choice in these architectures in the context of the task of deciphering the genetic code.

The weight adjustment is most beneficial to the simple fully linear MLP networks with respect to the required amount of data pairs in the training set, when the network actually has a relatively limited capacity. Indeed, when the size of the hidden layers is large, there is no benefit of weights adjustment. A recurrent neural network does not reduce much further the required amount of training data upon using amino acid weight adjustment.

## 4. Discussions

To our knowledge, cracking the genetic code using neural networks has never been done before or at least not been published. Our pedagogical showcase confirms that the genetic code can, indeed, be deciphered using a completely data-driven approach. Self-learning algorithms, when trained upon presentation of a large number of codon/amino acid pairs taken from pairs of transcripts with their associated proteins, can build the complete dictionary, correctly mapping the 64 codons to the 20 amino acid plus the stop mark.

Deep neural networks require large amount of data to train them. Deciphering the genetic code by a neural network is no exception. Two situations need to be distinguished: (i) if the network is shallow, i.e., with a small number of hidden layers and/or with a small capacity, the training is prone to have a poor data efficiency, meaning the required training data size will be large to achieve an arbitrarily given small loss. We showed that, with a shallow single hidden layer of width equal to 64, the minimal number of data pairs was in the range 12–22 million codon/amino acid pairs. The human transcriptome has around 73,000 transcripts and a total cumulated length of around 29 million codons. This means that a task as simple as deciphering the genetic code, resorting to a shallow neural networks required to use at least between 40 and 75% of the human transcriptome total information. If we had chosen a species with a much smaller genome or transcriptome, e.g., bacterium, a single species dataset would not have been sufficient. The union of several individual species datasets would have been necessary. On the contrary, (ii) if the network is deeper, i.e., with a larger number of hidden layers and/or with a higher capacity (width = 1, 024 instead of 64 for instance), the training is more data efficient and the minimal training data size to achieve the same result can be reduced. With the neural network settings we investigated in this study, the minimal number of required training data pairs to unequivocally crack the code was 4 million. So, the reduction in the training data size to decipher the genetic code was 80% with the deep neural network as compared to a shallow neural network.

For human beings, a deeper neural network may appear to be less comprehensible than a shallow one. Deciphering the genetic code is a fully linear problem as it comes down to simply determine a matrix ℝ^21 × 64^ projecting the space of codons to the space of amino acids. Intuitively, we would expect that a shallow neural network limited to a single layer implementing such a 21 by 64 matrix would be sufficient and would be data efficient. It works indeed but it is very far from the optimal data efficiency.

The lesson we can draw from this pedagogical showcase is that deeper neural networks of larger widths (capacity) will be prone to learn with less data. In the biomedical field, where biological data are sometimes very laborious or expensive to obtain, going for deeper neural network architectures is valuable even if it is at the cost of computer time complexity or even at the cost of losing direct human mind intuition or comprehension.

The use of the weights to adjust the penalization of the cross entropy loss to compensate for the class unbalanced distribution helped earlier decoding the rare amino acids by shallower neural network or with smaller capacity. The required training data size decreased by around 40%. This confirms a general rule in Statistics, that, the better the structure of the dataset is known, the more information can be retrieved from it or the less data is needed to retrieve the same amount of information. However, in this genetic table deciphering showcase, the data savings was the most significant upon resorting to deeper neural network than upon using the prior knowledge of the structure of the data.

The literature about embeddings schemes is numerous. In the field of natural language processing (NLP), text sequences with a similar linguistic contexts are identified by exploiting a so called word embedding. In the *word2vec* embedding approach, words or phrases are mapped to vectors of real numbers in a low dimensional space. By training a neural vector over a large text corpus, words with similar linguistic context correspond to vectors that are close points in the Euclidean space of the chosen dimensionality. In the field of Bioinformatics, *FastText* or *FastDNA* are extensions of *word2vec*, where instead of using individual words to train the neural network, words are broken into several n-grams used to train the network (Bojanowski et al., 2017; Menegaux and Vert, 2019). The identification of a similarity signature in a sequence exploited the Lyndon factorizations (Bonizzoni et al., 2021). It was used in the development of bijective Burrows-Wheeler Transform (Köppl et al., 2020). This latter algorithm is implemented in the alignment of overlapping reads of different lengths to be mapped on a given genome. *DNABERT* is another NLP model for DNA general embeddings (Ji et al., 2021). It uses the Bidirectional Encoder Representations from Transformers (BERT). Such transformers are relevant when context must be investigated on text sequences that are spatially or temporally further apart from each other. Recently, Bonizzoni et al. used some variants of the Lyndon factorization and developed an embedding features extraction method, *lyn2vec*, that is based on combinatorial properties providing compact embedding representations able to preserve similarities but without requiring a previous training (Bonizzoni et al., 2021, 2022).

Protein synthesis and mRNA translation can be directly related to the field of NLP and to the problem of language translation in general where the research literature is huge and still in fast development, especially with respect to theoretical developments in mathematical logic and to the introduction of new concepts in data processing such as RNN Turing complete theorem (Siegelmann and Sontag, 1991), Siegelmann and Sontag (1995), Carmantini et al. (2015), LSTM, encoder/decoder, variational auto-encoders (Kingma and Welling, 2019), and transformers (Wolf et al., 2020). One of the most recent and valuable breakthroughs in language processing was the introduction of the concept of *attention* to incorporate textual context in order to improve translation accuracy (Vaswani et al., 2017). In our project to decipher the genetic code, we used basic neural network architectures and did not require to capture the context of distant codon words or space correlation between them. The new contributions such as *attention* were not relevant for our problem. Contextual methods beyond the codon would be beneficial with longer space correlation within mRNA sequences incorporating untranslated regions (UTRs) or introns/exons. It should be noted that Machine Learning methods such as hidden Markov models are widely used in bioinformatics to infer whether or not a DNA sequence is coding a gene or not and to discriminate from non-coding DNA (Jones and Pevzner, 2004).

The showcase is an example of data-driven inference able to establish links (strong correlations) between two datasets but it did not unravel the detailed mechanisms by which a codon is actually translated into an amino acid, involving the full biochemical processes occurring in cells. Indeed, the neural network architectures we implemented did not possess the capacity to discover the transfer ribonucleic acids (tRNA) agents or the ribosome as the true causal biochemical links between codons and amino-acids. To make sense of a data-driven inferred knowledge, a mechanistic model can help to further unravel the underlying causal links. In a foreseeable future, the biomedical community will still have to investigate the underlying biochemical causes beyond the possible achievements of data-driven inference. There is an increasing trend in the biomedical sciences community to combine both mechanistic and data-driven technologies, depending on the availability of data and mechanistic knowledge (Viceconti et al., 2015; Eriksson et al., 2022).

We conclude that the wide generic capacities and modularity of deep neural networks allow them to be customized easily to learn the deciphering task of the genetic code efficiently. We meant to show that generic self-learning algorithms could learn and unravel the known genetic code scientific textbook knowledge by a completely data-driven approach. We also provided an idea of the amount of data required to train deep learning neural networks in the context of this specific task that could serve as a benchmark in the field of molecular biology. This showcase can be used by the biomedical community as a pedagogical example supporting the interest, possibilities and limitations of Artificial Intelligence in its research practice.

## Data availability statement

The datasets presented in this study can be found in online repositories. The names of the repository/repositories and accession number(s) can be found in the article/Supplementary material.

## Author contributions

MJ suggested the idea of applying neural networks to crack the genetic code, prepared and curated the datasets of the transcripts and proteins pairs used for training and testing, and produced the dynamic heatmaps (.gif and .mp4 videos in Supplementary material). GLo lectured and mentored MJ and ML in Deep Learning. GLo and GLa introduced the PyTorch modules to ML and MJ and supervised the general application of the Deep Learning methods that were used. MJ and ML wrote all python scripts using PyTorch and wrote the manuscript draft. GLa quality checked and corrected the python scripts, advised on the neural network architectures, and suggested the codon embedding layer to shed light on codon semantic similarity. All figures were produced by MJ. GLa, FR, PC, GLo, and LG proofread and corrected the manuscript draft. LG allocated time resources for MJ and funding. PC and FR allocated time resources for ML and funding. All authors are accountable for the content of the work. All authors contributed to the article and approved the submitted version.
